# Poster 1011: Potential immunogenetic associations between atopy and metabolic syndrome in children

**DOI:** 10.1186/1939-4551-7-S1-P6

**Published:** 2014-02-03

**Authors:** Damian Palafox, Brenda Tello-López, Laura Vidal

**Affiliations:** 1 Hospital General Gea Gonzalez. Mexico, Mexico; 2 Hospital Infantil de México Federico Gomez., Mexico; 3 Allergy and Immunology Department. Respiratory and Allergic Diseases Unit Xalapa, Mexico

## Background

Recently, immunogenetic studies have demonstrated a potential association between atopy and metabolic syndrome in children. Several susceptibility genes have been linked between obesity, diabetes and atopy, such as CTLA4 (CD28-ICOS), IL12b, ADRB2, TNFa and LTA4. It has also been noted that obese atopic patients have different clinical manifestation compared to those not obese, for instance, systemic inflammation is often more severe and bronchial hyperreactivity tends to be more acute. On the other hand, pulmonary function, quality of life in terms exercise tolerance and involvement in recreational activities is poorer.

## Methods

We herein present a 15 year old male atopic patient with a body mass index (BMI) of 28, with diagnosis of asthma, rhinitis and impaired glucose tolerance. Beta2-adrenergic agonist and metformin was administered with regression of mean glucose values to normal as well as control of pulmonary manifestations. Immunoglobulin determination was normal. Total White Blood Count, Lymphocytes, T helper Cells, Cytotoxic T cells, Natural Killer Cells and B cells count was normal. However, we tested for variations in the interleukin 4 receptor A (IL4RA) gene as it has been previously reported to be linked in asthma and type 1 diabetes and found no relation between them.

## Results

We further genotyped for single nucleotide polymorphisms (SNPs) in TLR2 and TLR4. TLR2 rs3804100 T allele was present in our patient. This SNP has been previously proposed as a possible susceptibility locus common for both Type 1 diabetes and asthma. We also found an increased expression of CCR2 and MMP9, which has been linked to BMI and homeostatic model assessment-insulin resistance. Certainly, pathogenic mechanisms are quite different in Type 1 and Type 2 diabetes, however, immunogenetic studies may demonstrate in the future a precise link between states of impaired glucose tolerance and atopy. For instance, in adults, Brumpton and colleagues have demosntrated that metabolic syndrome (high waist circumference, and elevated glucose or diabetes) has been pointed out as a risk factor for incident asthma.

## Conclusions

We consider that immunogenetic studies, including determination of HLA, SNPs and CNVs may provide information on the precise mechanism and link between childhood obesity and asthma. This studies, yet to come, are essential in countries such as Mexico and the US where Overweight is a very important public health issue.

